# Iatrogenic Kaposi Sarcoma Revealed by Acral Nodules With Silent Pulmonary Involvement in Two Patients Treated for Autoimmune Blistering Diseases

**DOI:** 10.7759/cureus.99633

**Published:** 2025-12-19

**Authors:** Youssef Zemmez, Adil Zegmout, Zakaria Toufgua, Rachid Frikh, Naoufal Hjira

**Affiliations:** 1 Dermatology Department, Mohamed V Teaching Military Hospital, Rabat, MAR; 2 Pulmonology Department, Mohamed V Teaching Military Hospital, Rabat, MAR; 3 Radiology Department, Mohamed V Teaching Military Hospital, Rabat, MAR

**Keywords:** corticosteroids, iatrogenic immunosuppression, kaposi sarcoma (ks), mtor inhibitors, pulmonary involvement

## Abstract

Iatrogenic Kaposi sarcoma (KS) is an uncommon but serious complication of prolonged immunosuppressive therapy. While most reported cases occur in transplant recipients, it may also develop in patients receiving corticosteroids for autoimmune dermatologic diseases. Early recognition is crucial, as presentation may be subtle and visceral involvement can occur without symptoms. We report two patients treated with prolonged systemic corticosteroids for bullous autoimmune dermatoses who developed isolated acral nodules on the toes. Both lesions were painless and clinically suggestive of vascular or melanocytic tumors. Histopathology confirmed Kaposi sarcoma with human herpesvirus-8 (HHV-8) positivity. Staging chest CT revealed asymptomatic bilateral pulmonary involvement in each patient. Corticosteroids were tapered and discontinued, and temsirolimus 25 mg IV weekly was initiated with antihistamine and dexamethasone premedication. Both patients demonstrated clinical stability and radiological persistence without progression at three-month follow-up. These cases illustrate that acral nodules may be the first sign of steroid-associated Kaposi sarcoma in non-transplant patients. Pulmonary dissemination may be silent, underscoring the importance of imaging. Mammalian target of rapamycin (mTOR) inhibition represents a relevant therapeutic strategy following the reduction of immunosuppression. Clinicians should maintain a high index of suspicion for Kaposi sarcoma in immunosuppressed patients presenting with new acral lesions. Prompt biopsy, staging, steroid tapering, and consideration of mTOR inhibitors may support disease control.

## Introduction

Kaposi sarcoma (KS) is a multifocal vascular neoplasm driven by human herpesvirus-8 (HHV-8), characterized by abnormal proliferation of endothelial-derived spindle cells forming angioproliferative lesions in the skin, mucosa, and visceral organs [1]. While its incidence has declined in high-income countries due to advances in human immunodeficiency virus (HIV) management, KS continues to pose a global health concern, particularly in regions with high HHV-8 endemicity [2]. Four clinicopathological subtypes are classically recognized: classic, endemic African, epidemic acquired immune deficiency syndrome (AIDS)-related, and iatrogenic KS, the latter being associated with immunosuppression in transplant recipients or patients treated for autoimmune and inflammatory conditions [3].

Iatrogenic KS was historically described in solid organ transplant recipients, but its epidemiology has shifted with the expanding use of long-term immunosuppressive therapy beyond the transplant setting [4]. Corticosteroids in particular exert profound effects on cellular immunity, facilitating HHV-8 reactivation, angiogenesis, and the proliferation of infected endothelial cells [5]. Cutaneous involvement predominates and typically affects the lower extremities; however, visceral dissemination, especially pulmonary involvement, may occur and significantly alter prognosis [6].

Although KS in transplant recipients has been well documented, reports involving non-transplant patients receiving prolonged corticosteroid therapy for autoimmune dermatologic diseases remain comparatively limited. Furthermore, distal acral presentations, such as isolated toe nodules, are exceedingly rare and may mimic benign or malignant cutaneous tumors, leading to diagnostic delay [7]. Importantly, pulmonary KS may remain asymptomatic and be detected only through systematic imaging, underscoring the need for a high index of suspicion in immunosuppressed individuals [8].

This gap in the literature highlights the importance of recognizing unusual cutaneous manifestations as potential harbingers of systemic disease in non-HIV, non-transplant immunosuppressed patients. Early dermatologic evaluation and timely radiological screening could be crucial for identifying subclinical visceral involvement and informing therapeutic decisions.

In this report, we present two cases of iatrogenic KS in patients receiving long-term corticosteroid therapy for autoimmune blistering dermatoses, both initially manifesting as isolated acral digital nodules and subsequently found to have asymptomatic pulmonary involvement. Acral nodular lesions in immunosuppressed patients may mimic a wide range of benign and malignant conditions, contributing to diagnostic uncertainty. These observations aim to raise clinical awareness of atypical presentations of iatrogenic KS and emphasize the value of prompt biopsy and systemic evaluation in this growing patient population.

## Case presentation

Case 1

**Figure 1:**
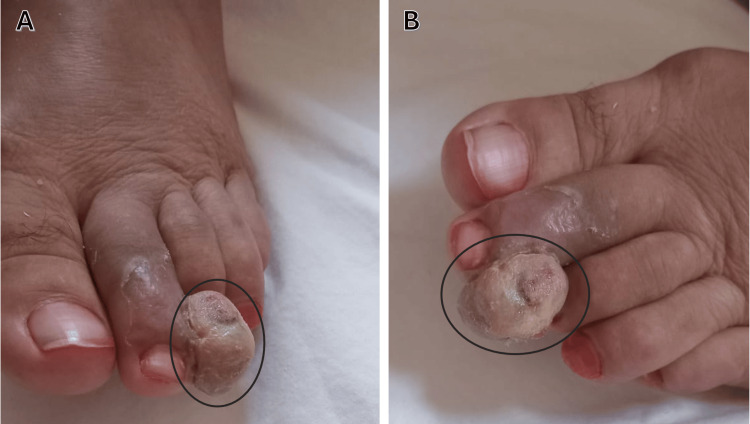
Case 1: Nodular lesion, soft and smooth-surfaced, partially exophytic and extending toward the third toe (circled). A: Dorsal view. B: Lateral view.

A 60-year-old woman was followed for bullous pemphigoid diagnosed four months earlier and initially treated with oral prednisone at 0.5 mg/kg/day for one month, followed by a progressive taper. She presented with a one-month history of a painless enlarging lesion on the distal aspect of the second left toe. No trauma, fever, weight loss, or respiratory symptoms were reported. Physical examination revealed a 15-mm violaceous nodular lesion, soft and smooth-surfaced, partially exophytic and extending toward the third toe, without ulceration or tenderness (Figure 1).

**Figure 2:**
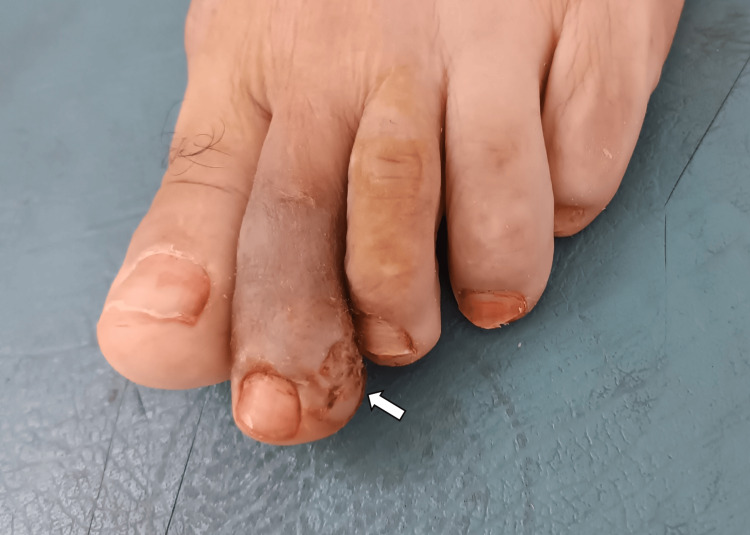
Case 1: Post-excisional view of the second toe showing the biopsy site (arrow) with a small ulcerated area.

No additional cutaneous or mucosal lesions or lymphadenopathy were present. Differential diagnoses included amelanotic melanoma, pyogenic granuloma, and keratoacanthoma. A complete excisional biopsy was performed (Figure 2).

**Figure 3:**
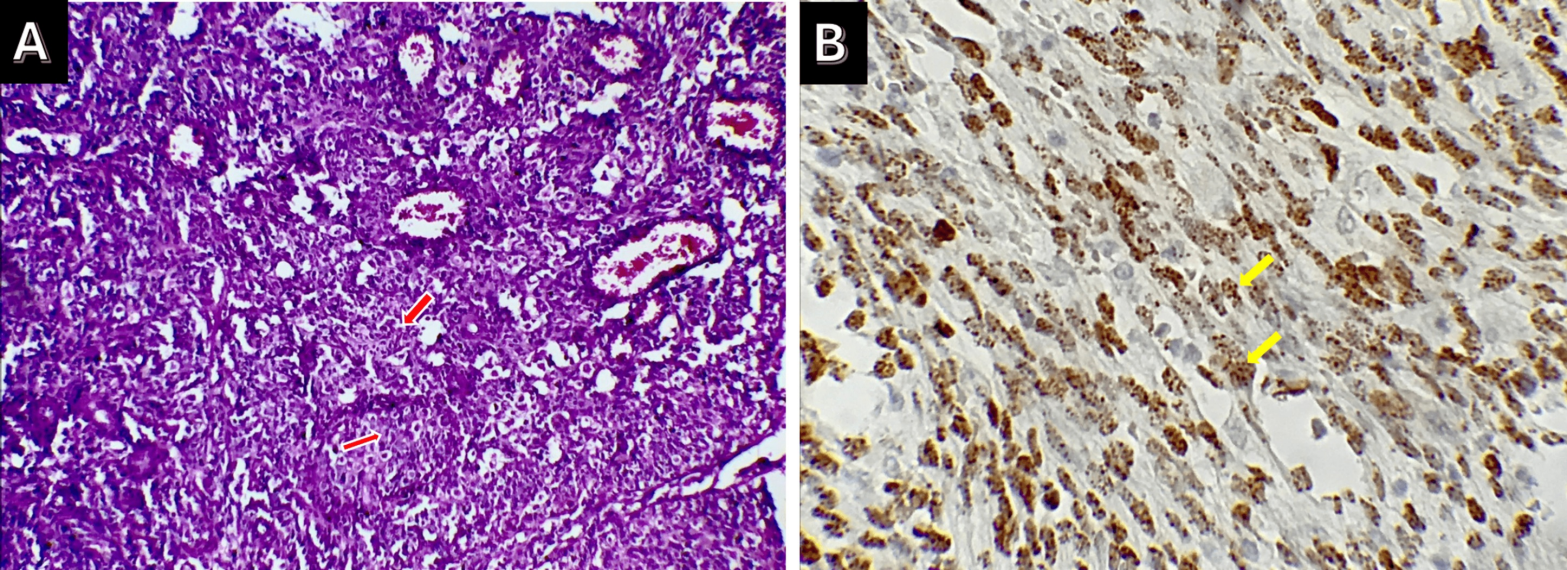
Case 1: A: Histological examination revealed a tumor proliferation made up of spindle cells (red arrows) organized in short intersecting bundles. B: The immunohistochemical study revealed positive CD34 (yellow arrows) and HHV-8 staining, confirming the diagnosis of Kaposi sarcoma. HHV-8: human herpesvirus-8

Histopathology showed a spindle cell proliferation arranged in intersecting fascicles with slit-like vascular spaces and extravasated erythrocytes (Figure 3A), with immunohistochemical expression of CD34 and HHV-8, confirming Kaposi sarcoma (Figure 3B).

**Figure 4:**
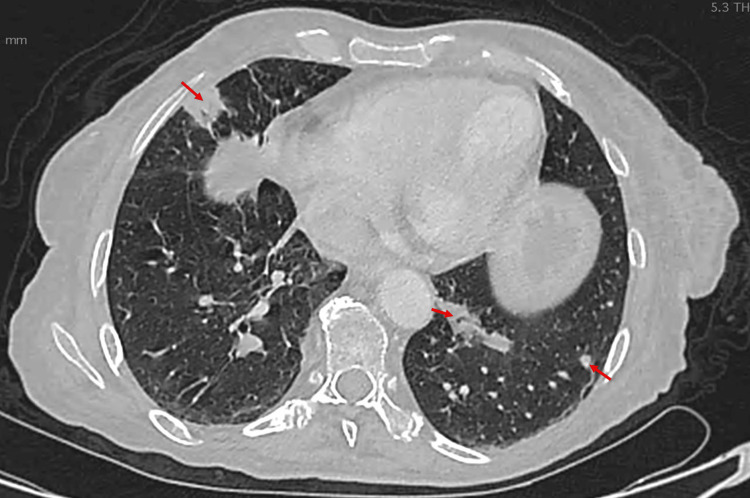
Case 1: Axial chest CT demonstrating multiple bilateral pulmonary nodules (arrows) with peribronchovascular and peripheral distribution.

A chest CT scan demonstrated multiple bilateral pulmonary nodules with peribronchovascular and peripheral distribution and moderate interstitial changes, without pleural effusion or mediastinal lymphadenopathy (Figure 4). The patient remained asymptomatic.

Biology was unremarkable: aspartate aminotransferase (ASAT), 24 UI/L; alanine aminotransferase (ALAT), 19 UI/L; creatinine, 8 mg/L; and urea, 0.32 g/L. Her immunological profile was as follows: lymphocyte count, 1,250/mm³, and CD4 count, 410/mm³. Her HIV, hepatitis B virus (HBV), and hepatitis C virus (HCV) serologies were negative, and her HHV-8 serology was positive.

Corticosteroids were tapered and discontinued. Temsirolimus 25 mg IV weekly was initiated with antihistamine and dexamethasone premedication. Treatment was well tolerated. At the three-month evaluation, no new lesions were observed, and repeat chest CT showed persistent but stable pulmonary nodules. The patient remained clinically stable, without new cutaneous or visceral involvement.

Case 2

**Figure 5:**
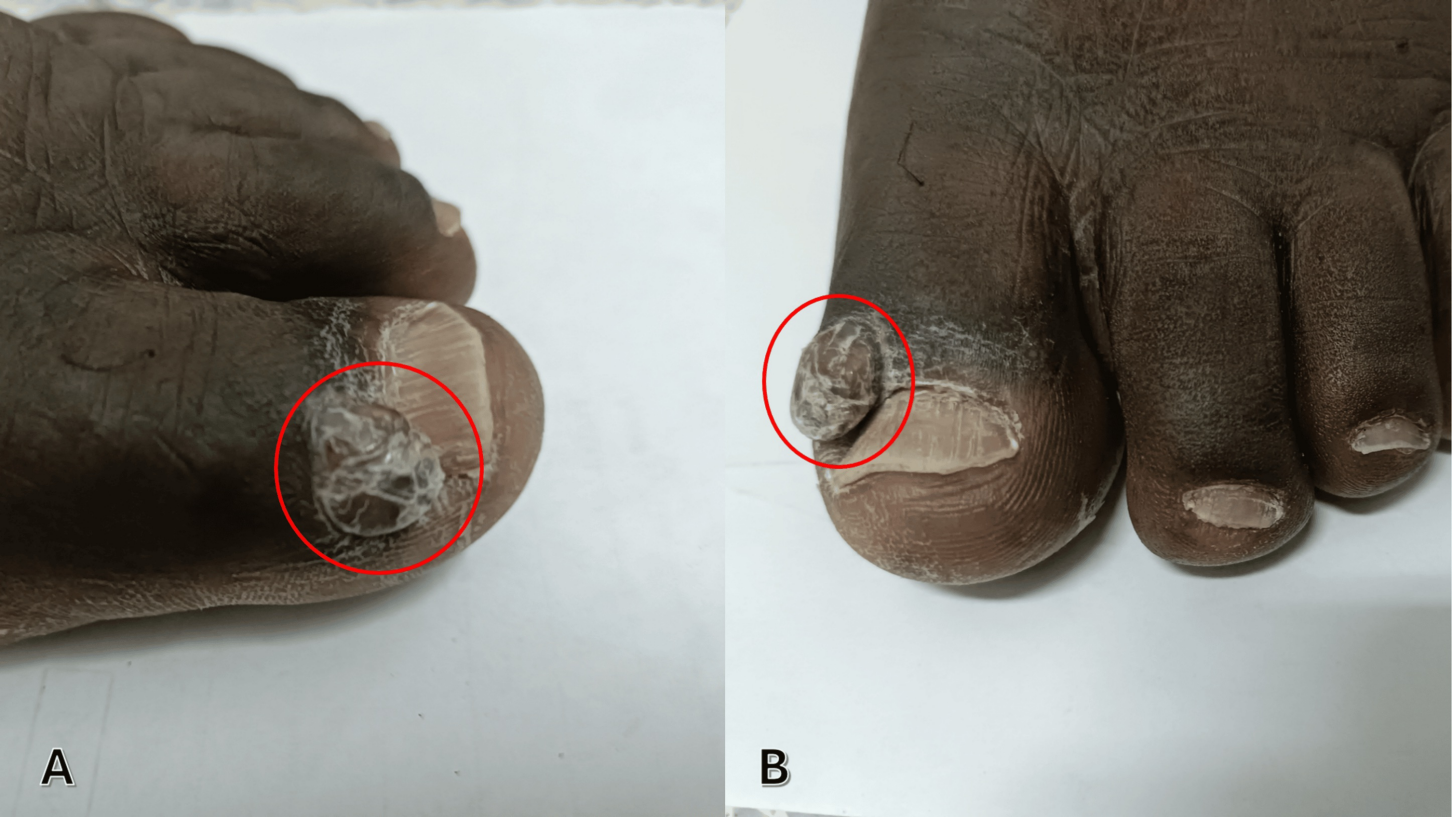
Case 2: Nodular formation at the tip of the left big toe (circled). A: Lateral view. B: Dorsal view.

A 52-year-old man with superficial seborrheic pemphigus, treated with oral prednisone 1 mg/kg/day for six months, presented with a two-month history of a painless erythematous nodule on the lateral aspect of the left great toe. No systemic or respiratory symptoms were reported. Examination revealed a 10-mm angiomatous soft nodule with a smooth surface, non-tender and non-ulcerated (Figure 5).

**Figure 6:**
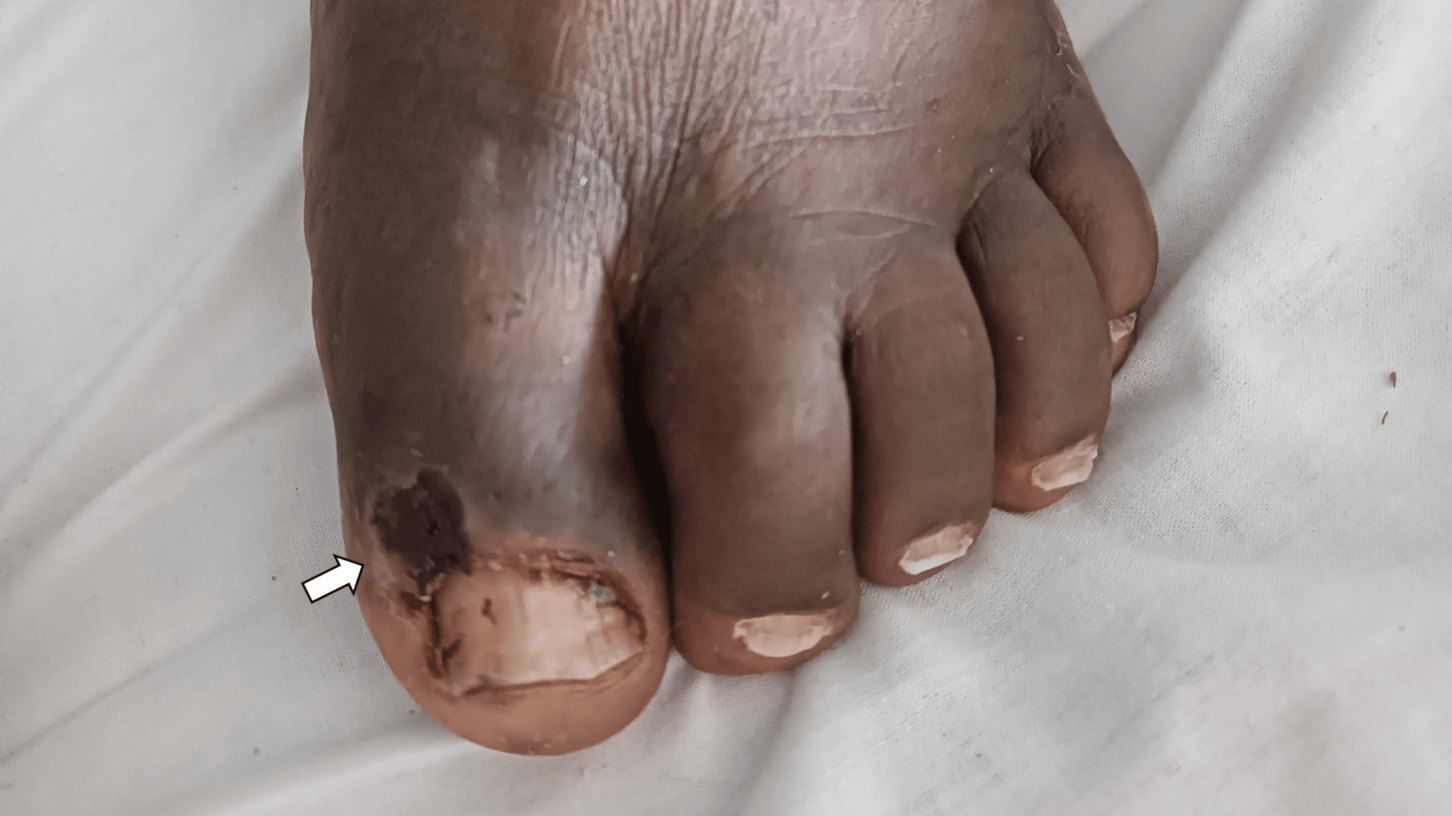
Case 2: Post-excisional view of the left big toe showing the biopsy site (arrow) with a small ulcerated area.

No mucosal lesions or lymphadenopathy were present. Differential diagnoses included amelanotic melanoma, botryomycoma, and fibrokeratoma. An excisional biopsy was performed (Figure 6).

**Figure 7:**
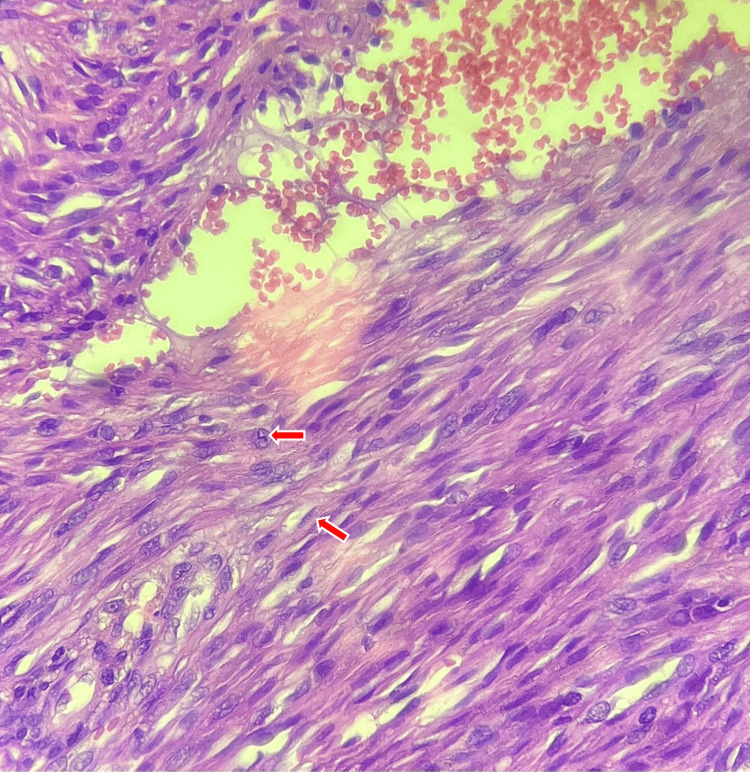
Case 2: Tumor proliferation consisting of spindle cells (arrows) organized in short, intertwined bundles (H&E, 300×). H&E: hematoxylin and eosin

Histopathology showed a fusiform spindle cell proliferation with slit-like vascular spaces, showing immunohistochemical positivity for CD34 and HHV-8, consistent with Kaposi sarcoma (Figure 7).

**Figure 8:**
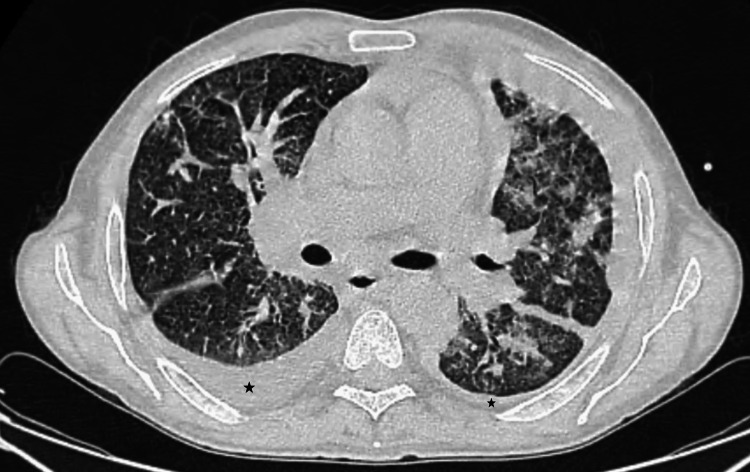
Case 2: Axial slice of a chest CT scan demonstrating bilateral interstitial involvement with septal thickening, micronodules of mixed distribution, and bilateral pleural effusion (stars).

A thoraco-abdominopelvic CT showed bilateral interstitial involvement with septal thickening, mixed-distribution micronodules, and bilateral pleural effusion (Figure 8). The patient remained asymptomatic.

Laboratory results were normal: ASAT, 22 UI/L; ALAT, 21 UI/L; creatinine, 9 mg/L; and urea, 0.35 g/L. His immunological profile was as follows: lymphocyte count, 1,180/mm³, and CD4 count, 395/mm³. His HIV, HBV, and HCV serologies were negative, and his HHV-8 serology was positive.

Corticosteroids were tapered. Temsirolimus 25 mg IV weekly was initiated with antihistamine and dexamethasone premedication. Tolerance was good. At the three-month follow-up, chest CT demonstrated no new lesions and stable pulmonary findings, without recurrence of cutaneous disease or clinical deterioration.

## Discussion

Kaposi sarcoma (KS) is a multifocal vascular neoplasm driven by HHV-8 infection, with the iatrogenic form most often described in solid organ transplant recipients and, less frequently, in patients receiving prolonged immunosuppressive therapies for autoimmune diseases. Chronic impairment of T-cell-mediated immunity facilitates HHV-8 reactivation and endothelial proliferation, ultimately promoting tumorigenesis [1,3]. Steroid-induced KS has been reported across dermatologic, rheumatologic, and gastrointestinal disorders, yet remains uncommon and likely under-recognized outside transplant medicine [7,9].

The cases presented here highlight several distinctive clinical aspects of iatrogenic KS in non-transplant settings. Both patients initially developed acral digital nodules, an unusual initial manifestation that may mimic other vascular or melanocytic tumors. Dermoscopy may represent a useful adjunctive tool in the evaluation of acral nodular lesions, particularly in immunosuppressed patients. Although the rainbow or polychromatic pattern has traditionally been considered suggestive of Kaposi sarcoma, it has also been described in a wide range of non-Kaposi conditions, including benign vascular tumors such as pyogenic granuloma and angiokeratoma, cutaneous malignancies such as basal cell carcinoma, melanoma, and Merkel cell carcinoma, and inflammatory dermatoses and scars. This pattern is thought to result from an optical phenomenon related to the interaction of polarized light with tissues rich in vascularization, fibrous components, and pigments, particularly in nodular, deep, and acral lesions. Consequently, while dermoscopy can help guide diagnostic orientation, it does not reliably distinguish Kaposi sarcoma from its mimickers, making histopathological confirmation mandatory [10].

Acral involvement has been reported in a minority of iatrogenic KS cases and is associated with diagnostic delay, particularly in asymptomatic patients [4,11]. In both patients, pulmonary involvement was detected on staging CT, despite the absence of respiratory symptoms. Radiologically, pulmonary Kaposi sarcoma typically presents on chest CT as bilateral peribronchovascular and subpleural nodules, interstitial thickening, septal lines, and, in some cases, pleural effusions, reflecting lymphatic and vascular involvement [5]. These findings, although not specific, should raise suspicion in the appropriate clinical and immunological context. Pulmonary KS may be clinically silent and is often diagnosed at an advanced stage, emphasizing the importance of systematic thoracic imaging when KS is suspected in immunosuppressed patients [5,6].

Glucocorticoids play a pivotal role in KS pathogenesis by inhibiting T-cell immunity and acting directly on HHV-8-infected endothelial cells through glucocorticoid-responsive promoter activation, thereby promoting viral replication and angiogenesis [7]. In both cases, KS appeared under prolonged systemic corticosteroid therapy for autoimmune blistering diseases, supporting previous observations that steroid therapy can serve as a triggering factor in susceptible hosts. Importantly, lesion stabilization and regression occurred following corticosteroid withdrawal, consistent with prior reports [8,9].

Mammalian target of rapamycin (mTOR) inhibition has demonstrated efficacy in transplant-related KS, where conversion from calcineurin inhibitors to sirolimus results in lesion regression [9]. Temsirolimus, although less frequently reported, shares similar mechanisms and has shown encouraging responses in HHV-8-associated malignancies [12]. The favorable tolerance and partial regression observed in our patients support mTOR pathway modulation as a therapeutic strategy in selected cases of steroid-associated KS outside the transplant context.

Our observations shed new light on the evolving clinical spectrum of iatrogenic Kaposi sarcoma. In particular, they show that disease onset may be deceptively subtle, beginning with painless acral nodules in patients receiving immunosuppressive therapy for autoimmune conditions. Such cutaneous manifestations, frequently overlooked or misinterpreted as benign vascular lesions, warrant heightened clinical suspicion even in the absence of systemic symptoms.

Equally noteworthy, both patients exhibited silent pulmonary involvement, demonstrating that visceral dissemination can occur early and asymptomatically in non-HIV, non-transplant settings. This finding underscores the need for systematic thoracic imaging in the initial workup of suspected Kaposi sarcoma in immunosuppressed individuals, irrespective of respiratory complaints.

From a therapeutic standpoint, corticosteroid tapering remains essential; however, it may not achieve disease control on its own. The favorable disease stabilization observed with temsirolimus in our patients supports the early consideration of mTOR inhibitors in selected cases, particularly when systemic spread is suspected or confirmed.

Taken together, these cases emphasize the importance of a multidisciplinary approach. Close collaboration between dermatologists, pathologists, infectious disease specialists, and pulmonologists facilitates timely diagnosis, appropriate staging, and individualized treatment strategies. Above all, they remind clinicians that even isolated acral lesions in immunosuppressed patients may herald more extensive visceral disease and that early, coordinated intervention can help prevent progression.

## Conclusions

Iatrogenic Kaposi sarcoma remains a rare but important complication of prolonged immunosuppressive therapy for autoimmune dermatologic disorders. These cases demonstrate that isolated acral nodules may represent the first clinical manifestation, even in the absence of systemic symptoms, and should prompt urgent histopathological evaluation. Systematic staging, including chest CT, is essential given the potential for silent pulmonary involvement. In both patients, tapering corticosteroids combined with temsirolimus was associated with clinical stability and radiological disease control at three months. These observations highlight the importance of early recognition, multidisciplinary evaluation, and individualized immunomodulatory strategies in the management of steroid-associated Kaposi sarcoma.
